# Screening and function discussion of a hereditary renal tubular acidosis family pathogenic gene

**DOI:** 10.1038/s41419-020-2354-y

**Published:** 2020-03-02

**Authors:** Li Chen, Han-Lu Wang, Yao-Bin Zhu, Zhao Jin, Jian-Bin Huang, Xin-Fu Lin, Jie-Wei Luo, Zhu-Ting Fang

**Affiliations:** 1 Shengli Clinical Medical College of Fujian Medical University, Fuzhou, 350001 China; 2 Department of Traditional Chinese Medicine, Fujian Provincial Hospital, Fuzhou, 350001 China; 3 Department of Traditional Chinese Medicine, the First Affiliated Hospital, Fujian Medical University, Fuzhou, 350005 China; 4 Department of Paediatrics, Fujian Provincial Hospital, Fuzhou, 350001 China; 5 Department of Interventional Radiology, Fujian Provincial Hospital, Fuzhou, 350001 China

**Keywords:** DNA damage and repair, DNA damage and repair, Kidney diseases, Kidney diseases

## Abstract

Hereditary distal renal tubular acidosis (dRTA) is a rare disease of H^+^ excretion defect of α-intercalated cells in renal collecting duct, caused by decreased V-ATPase function due to mutations in the *ATP6V1B1* or *ATP6V0A4* genes. In the present study, a genetic family with 5 members of the complete dRTA phenotype were found with distal tubule H^+^ secretion disorder, hypokalemia, osteoporosis, and kidney stones. A variant NM_020632.2:c.1631C > T (p.Ser544Leu) in exon 16 on an *ATP6V0A4* gene associated with dRTA was detected by next generation sequencing target region capture technique and verified by Sanger sequencing, which suggested that except for one of the patients who did not receive the test, the other four patients all carried the p.S544L heterozygote. In transfected HEK293T cells, cells carrying p.S544L-mut showed early weaker ATPase activity and a slower Phi recovery rate after rapid acidification. By immunofluorescence localization, it was observed that the expression level of p.S544L-mut on the cell membrane increased and the distribution was uneven. Co-immunoprecipitation showed the a4 subunit of *ATP6V0A4/*p.S544L-mut could not bind to the B1 subunit, which might affect the correct assembly of V-ATPase. The present study of dRTA family suggests that the p.S544L variant may be inherited in a dominant manner.

## Introduction

Renal tubular acidosis (RTA) is a clinical syndrome characterized by normal anion gap (AG) and high chloride metabolic acidosis caused by renal acidification dysfunction. It could be caused by H^+^ obstruction of distal renal tubules, or proximal renal tubules for bicarbonate (HCO_3_^−^) reabsorption, or both. Clinically, it is divided into four types: distal RTA (type I, dRTA), proximal RTA (type II, pRTA), mixed RTA (type III), hyperkalemic RTA (type IV)^1–3^. The etiologies of RTA might be primary tubular disease, systemic disease, or side effects of medicine. Most of the primary defects are congenital defects of the renal tubules and often related to heredity; secondary diseases could be seen in many diseases, mainly are autoimmune diseases such as Sjogren’s syndrome, systemic lupus erythematosus^3–7^. dRTA is defined as the disease caused by distal renal tubular H^+^ secretion disorder, reduced discharge of urine NH_4_^+^ and titratable acid. Urine acidification dysfunction means that the pH could not be reduced to 5.3–5.5 in the case of systemic metabolic acidosis, which is clinically common^7,8^. Hereditary dRTA is a rare disease caused by genetic mutation resulting in H^+^ excretion defect of α-intercalated cells (α-ICs) in renal collecting duct. Its main clinical features are persistent normal AG hyperchloric metabolic acidosis, hypokalemia, osteoporosis, and renal calcinosis^9^. The present study describes a clinical phenotype and genetic responsibility variant screening for a dRTA family, followed by functional identification.

## Materials and methods

### Research objects

**Fig. 1:**
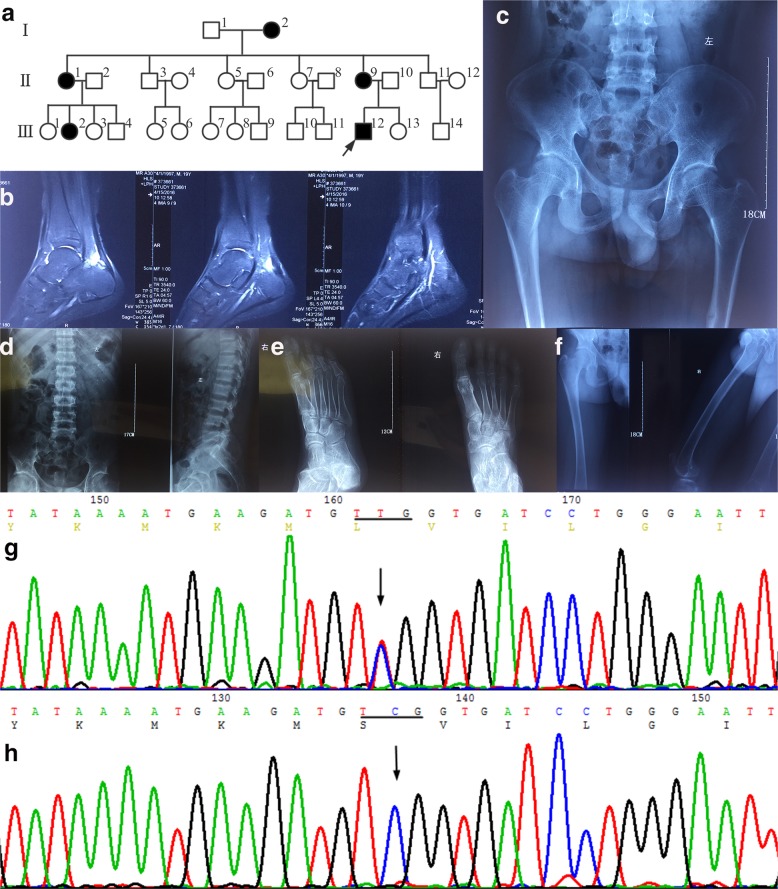
The pedigree chart, phenotype and genetic analysis of the family members. **a** Family diagram of hereditary renal tubular acidosis. Five patients were found in the family. Four patients who were sequenced all carried *ATP6V0A4*/p.Ser544Leu heterozygotes. **b** Right calcaneus MRI showed a small amount of effusion in the right ankle joint, and bursitis of the anterior Achilles tendon and Achilles tendinitis. **c**–**f** X-ray of pelvic, lumbar vertebrae, bilateral heel axis, and extremities showed osteoporosis features, considered as metabolic osteodystrophia. **g** A missense variant heterozygote of c.1631C > T(p.Ser544Leu) found in the proband on exon16 of *ATP6V0A4* (NM-020632.2) associated with dRTA. **h** c.1631C (p.Ser544) wild homozygotes.

Proband (III12, Fig. 1a): male, 19 years old, Chinese Han, chief complaint was “recurring generalized fatigue for 5 years”, and denied numbness of limbs, convulsions, urine volume abnormality, abnormal development, and hearing loss. The serum potassium level was 2.2–2.8 mmol/L checked at local hospital, normal blood pressure, symptoms could be alleviated after oral potassium chloride treatment, serum potassium could raise to the normal range when reexamined. Two years before admitted to the hospital, the patient often felt pain in the right heel after standing for a long time. Magnetic resonance imaging of the right calcaneus (Fig. 1b) examined in the local hospital showed a small amount of fluid in right ankle joint, bursitis and Achilles tendinitis in right ankle anterior Achilles tendon. The patient felt general weakness three days before admission. Serum potassium was 2.8 mmol/L and chlorine was 112 mmol/L examined in the local hospital. Symptoms were relieved after intravenous infusion of potassium chloride. For further diagnosis and treatment, the patient was referred to our hospital. The patient was the first child of the first-time pregnancy and denied that his parents were close relatives. After admission, a series of examinations such as electrocardiogram, chest and pelvic radiographs, and arterial blood gas analysis were performed on the proband. A family survey was conducted and a hereditary pedigree was made (Fig. 1a), finding five family members with similar medical history. Ammonium chloride (NH_4_Cl) loading test was performed on four patients. The study was approved by the Ethics Committee of the Fujian Provincial Hospital and all members of the family surveyed signed an informed consent form.

### Candidate gene mapping and mutation screening strategy

Peripheral blood of the proband and a part of family members were collected with an ethylenediaminetetraacetic acid anticoagulant tube. The peripheral blood DNA was extracted according to the instructions of QIAamp DNA Blood Mini Kit (QIAGEN, Cat No. 51106) and purified for subsequent experiment. The purity of the extracted DNA was determined using a NanoDrop^®^ instrument. The OD260/OD280 ratio was maintained at 1.8–2.0 to meet the subsequent sequencing process. At first, DNA whole genome library preparation, capture of target gene regions was performed. Next, sequencing was performed using a sequencing panel on the Illumina NextSeq 500 platform. This panel could be used to perform parallel analysis of multiple genes. The target genes involved are: *SLC4A1*, *ATP6V0A4*, *CA2*, *SLC4A4*, and ATP6V1B1. The sequencing data including all the SNVs and small indels were analyzed using ANNOVAR software^10^, and several databases, including 1000G, ESP6500, dbSNP, and HGMD were used according to the ACMG guidelines^11^ to filter and annotate variants. The reads obtained from were filtered, separated, and BWA and hg19 sequences were used for reference aligned to acknowledge important information about the variants, such as gene locus, variant type, 1000G and ESP6500 frequencies. Sorting intolerant from tolerant (SIFT, http://sift.jcvi.org/), polymorphism phenotyping (PolyPhen-2, http://genetics.bwh.harvard.edu/ppH2/), and mutation taster (http://mutationtaster.org/) were used to predict the pathogenicity of the disease to obtain gene mutation information and its biological significance. Primers 5.0 were used to design primers for the position of the suspect variant site (http://sg.idtDNA.com/Primerquest/Home/Index) and the target region was amplified. Sanger sequencing was performed on the ABI 3500 Dx platform to verify Illumina NextSeq 500 sequencing results. For example, the polymerase chain reaction (PCR) amplified fragment of the target sequence of NM_020632.2:c.1631C > T (p.Ser544Leu) of the suspicious variant *ATP6V0A4* was 274 bp, the primer was F: CCAAACCAGTGGCTCTGTCA; R: GTTGTGCTGTAGCCCTCAACT, and the annealing temperature was 62 °C. The primers were synthesized by Suzhou Synbio Technology Co., Ltd.

### Construction and transfection of *ATP6V0A4* gene p.S544L wild and mutant eukaryotic plasmids

The plasmids construction of the fragment in which p.S544L is located was constructed by gene synthesis. Primers were designed according to this principle: each primer must carry the desired variant site and the designed variant site should be located in the center of the primer. Primers: A4 Mut-F: CTCGTATAAAATGAAGATGTTGGTGATCCTGGGAATTGTCC; A4 Mut-R: GGACAATTCCCAGGATCACCAACATCTTCATTTTATACGAG; high-fidelity primer star polymerase was used and followed by 18 cycles of PCR reaction. EcoRI and BamHI in the vector plasmid pEGFP-Nl were selected as the restriction sites. After PCR purification, the target fragments were ligated with pJet1.2 vector (Xinyu, Shanghai, China) by using T4 DNA ligase (Thermo Fisher) to obtain a large number of intermediate plasmids of the desired fragment. The competent *Escherichia coli* cell DH5a strain was prepared through the CaCl_2_ method to express foreign genes. After the enzyme ligated products were transformed into the competent *E. coli* DH5a strain, then *E. coli* was coated on a medium containing the corresponding antibiotic to select the mutant type. If the foreign plasmid DNA is successfully transformed into *E. coli*, it could be grown on a medium containing an antibiotic (ampicillin sodium, Shanghai Biotech, China). Afterwards, a large number of recombinant plasmids were cloned by expanding the culturation of *E. coli*. A small amount of plasmid DNA was extracted and verified by sequencing. HEK293T cells were cultured in DMEM medium with high glucose + 10% fetal bovine serum + 1% P/S (Hyclone medium, SH30022.01B), and transfected with plasmids (TurboFect transfection reagent, Thermo, R0531). Total RNAs were extracted from the cultured cells according to the instructions of the Roche reagent kit Tripure isolation reagent (ROCHE, 11667165001). In addition, after the *ATP6V1B1* overexpression plasmid was biosynthesized, the CDS region of *ATP6V1B1* was constructed into the pBobi-N-3HA vector.

### Protein extraction and western blot analysis

A 20 mg of cell tissue was lysed by RAPI lysate to extract total protein. The protein concentration was determined by the bicinchoninic acid assay (BCA) method (BCA Protein Quantification Kit, Biyuntian, P0011), the absorbance at 562 nm was measured, and the protein concentration of the sample was calculated according to the standard curve. Equal amounts of protein were loaded per lane using 10% sodium dodecyl sulfate (SDS)-polyacrylamide gel electrophoresis, transferred onto polyvinylidene fluoride membranes and blocked for 1 h at room temperature in 5% skim milk prepared in TBST. The membrane was cut as needed and immersed in the prepared primary antibody solution at the dilutions recommended by the manufacturer, incubated overnight at 4 °C. Next, the membrane was incubated with secondary antibody which was selected according to primary antibody and diluted at 1:5000 at room temperature for 1 h, and ECL reagent was added to visualize the immunostained proteins.

### Immunofluorescence localization

The fixed cells were stored in phosphate-buffered saline (PBS) containing Sodium azide at 4 °C for 3 months. After washing with PBS, the cells were blocked using blocking solution for 30 min. Anti-flag (1:500, sigma, F2555) primary antibody and anti-Ms-488 (1:1000, Jackson, 209-545-082) secondary antibody were added into the cells, respectively. The nuclei were stained with DAPI and incubated for 1 h in the dark. High-sensitivity laser confocal microscopy (Zeiss, LSM780) was used to observe the cells after mounting.

### Co-immunoprecipitation (Co-IP)

Co-IP was performed with HA antibody and Flag antibody respectively, and 5% input sample was detected using tubulin as an internal reference and green fluorescent protein (GFP) as an external reference. After 10 μg of the plasmids were transfected by groups, 2 μg of the GFP control plasmids were added to 1000 μL of Opti-Medium and mixed into a TurboFect-DNA mixture. Afterwards, 20 μL of TurboFect was added and the mixed solutions was added dropwise to a single layer of HEK293T cells. After 48 h of transfection, the cells were harvested, lysed on ice, centrifuged at 4 °C, 15,000 g for 15 min, and the supernatant was stored at −20 °C. Totally, 5 μL of whole-cell lysates was taken as an input (5%). Totally, 100 μL protein samples were, respectively, added with 1 μL HA antibody, Flag antibody and 5 μL protein A/G magnetic beads for reaction overnight at 4 °C. The Ep tube was placed on the magnetic stand, the supernatant was removed and 1 mL of protein lysate was added. The mixed solution was shaken and placed on the mixer for 5 min which was repeated for 4 times. The sample was prepared by adding 30 μL of 1× SDS loading buffer into solution, and the Western Blot analysis was performed along with the input. Primary anti-mouse Monoclonal ANTI-FLAG^®^ M2 antibody (SIGMA, F3165), Anti-HA tag (Abcam, ab9110), mouse monoclonal anti-GFP antibody (SIGMA, SAB-2702197), tubulin antibody (Abcam, ab7291) were diluted with the proportion of 1:1000, 1:1000, 1:1000, 1:2000 with blocking solution. The secondary anti-Goat anti Mouse IgG (Thermo, 31430) was diluted with the proportion of 1:2000 and incubated for 1 h at room temperature on a shaker.

### Determination of ATPase activity and ATPase hydrogen ion transport function in transfected cells

The hydrolysis of ATPase producing ADP is accompanied by oxidation reaction of reduced nicotinamide adenine dinucleotide (NADH). Therefore, the unit enzyme activity could be measured by determining the change in the absorbance peak (NADH concentration) at 340 nm after oxidation by spectrometry. The co-immunoprecipitate product was mixed with 1× ATPase reaction buffer for 25 min and the change in concentration of NADH was determined. The Vacuolar (H^+^)-ATPase (V-ATPase) mediated hydrogen ion transport function was determined by fluorescence spectrophotometer (Thermo) method: The HEK293T cells expressing *ATPVOA4*-WT and *ATPVOA4*-mut proteins in a single layer were rapidly acidified using NH_4_Cl, and the transport function of ATPase hydrogen ions was measured by calculating the recovery rate of PH of cells that was independent on sodium transport. The research tool of this experiment was a multifunction microplate reader (Thermo, VARIOSKAN LUX).

### Statistics

The experimental data was statistically processed using graphpad Prism 6.02. The mean was expressed as mean ± SEM. The mean of multiple groups was compared by one-way analysis of variance. *P* < 0.05 was considered as statistically significant.

## Results

### Clinical data survey of RTA family

Proband (III12), male, 19 years old, chronic hypokalemia. The clinical manifestations were hypodynamia and lower hypokalemia under repeated stress, and serum potassium fluctuated at 2.2–2.8 mmol/L. After admission, the biochemical indicators of the probands (Table 1) showed serum potassium 2.74 mmol/L, serum phosphorus 0.66 mmol/L, serum chlorine 110 mmol/L, alkaline phosphatase 329 U/L, normal serum sodium and calcium, suggested hypophosphatemia, hyperchloremia, and hypokalemia. Various tumor markers (AFP, CEA, CA125, CA199, and CA153) are normal. Bone density measurement showed that the bone density of the left forearm was 0.236 g/cm^2^, which was five standard deviation lower than the peak bone mass of the same sex and five standard deviation lower than that of the same age, suggesting osteoporosis. X-ray of the frontal position of pelvis, lumbar vertebrae, and lateral position of both heel axes (Fig. 1c–e) showed that reduced bone mineral density, the trabecular bone was sparse, the cortical bone was thickened, blurred, and the density was uneven, the trabecular bone was rough and fuzzy, which might be considered as metabolic bone malnutrition. X-ray of limbs (Fig. 1f) showed that the femur and tibia diaphyses flexion deformities, metaphyses were slightly enlarged, the cortical bone was thin, the trabecular bone was irregular and fuzzy, and several transverse growth barriers were visible in the lower part of the tibia, calcaneus bone spur was formed. 24 h: urinary potassium 72.2 mmol/L (reference value 25.0–125.0 mmol/L), urinary sodium 127 mmol/L (reference value 40.0–220.0 mmol/L), urinary calcium 6.5 mmol/L (reference value 2.5–7.5 mmol/L), urinary chlorine 124 mmol/L (reference value 170–250 mmol/L). Blood gas analysis showed PH 7.333, PaCO_2_ 36.4 mmHg, PaO_2_ 94.8 mmHg, Hct 55%, Hb 179 g/L, extracellular residual base −6.0 mmol/L, residual base −5.9 mmol/L, standard bicarbonate (SB) 119.7 mmol/L, actual bicarbonate (AB) HCO_3_^−^ 18.8 mmol/L, total carbon dioxide 19.9 mmol/L, and lactic acid 2.0 mmol/L. Blood AG 15.2 mmol/L, suggesting metabolic acidosis, the proband (III12) could be diagnosed as RTA. The other four members of the family (Fig. 1a) (I2, II1, II9, and III2) also have different degrees of clinical symptoms such as fatigue, anorexia, kidney stones, or hypokalemia. Detailed clinical data and blood samples of the proband’s grandmother (I2) were not collected. Family members had no growth retardation, mental retardation, facial deformity, dental caries, mental illness, and no deafness, eye disease, or congenital heart disease. All the four diseased members including the proband, had different degrees of hypokalemia. After the ammonium chloride load test, the urine pH of II1, II5, III2, and III12 (proband) were greater than 5.3. Therefore, the family was considered as a hereditary dRTA family. In the ammonium chloride load test, it is a kind of test to check the function of distal renal tubular, urine pH > 5.3 after acidification indicates positive, which means that distal renal tubular acidification dysfunction caused type I RTA.

**Table 1:** Clinical data of eight members in hereditary renal tubular acidosis family.

Project	III_12_ (Proband)	II_1_	II_9_ (Proband’s mother)	III_2_	II_5_	III_7_	II_10_ (Proband’s father)	III_13_	Normal reference range
Gender	Male	Female	Female	Female	Female	Female	Male	Female	–
Age	19	52	41	29	47	22	43	17	–
Urine PH	8.0↑	7.5↑	8.0↑	7.4↑	6.3	6.0	6.5	6.5	4.8–7.4
Arterial blood PH	7.33↓	7.32↓	7.31↓	7.34↓	7.39	7.41	7.40	7.41	7.35–7.45
Arterial blood HCO_3_^−^ (mmol/L)	18.8↓	18.4↓	17.0↓	18.6↓	23.5	22.8	25.3	24.9	21.4–27.3
Plasma anion gap (mmol/L)	15.2	14.5	13.6	14.1	13.9	14.0	14.8	13.7	8–16
Serum K^+^ concentration (mmol/L)	2.74↓	3.0↓	2.22↓	2.9↓	3.91	3.92	4.20	4.01	3.5–5.1
Serum Cl^−^ concentration (mmol/L)	110↑	109↑	112↑	110↑	100	105	101	105	98–107
Serum Ca^2+^ concentration (mmol/L)	2.22	2.28	2.05↓	2.23	2.19	2.31	2.31	2.53	2.15–2.7
Serum phosphorus (mmol/L)	0.66↓	0.83↓	0.84↓	0.80↓	0.90	1.34	1.13	1.81↑	0.87–1.45
Serum creatinine Cr(μmol/l)	122	80	77.4	94.2	70	59.7	68.6	70	44–135
Red blood cell (×10^12^/L)	6.57↑	5.5	5.7	4.9	4.9	5.1	4.6	5.3	4.0–5.5
Hemoglobin (g/L)	190↑	138	127	154	128	153	132	161↑	120–160
Urine specific gravity	1.008	1.020	1.010	1.014	1.009	1.011	1.005	1.020	1.003–1.030
Growth and development	Normal	Normal	Normal	Normal	Normal	Normal	Normal	Normal	–
Osteopathy	Osteoporosis	No	No	No	No	No	No	No	–
Renal calculi	Double renal crystallization	Renal calculi	Double renal calculi	Renal calculi	No	No	No	No	–
NH_4_Cl load test^Δ^	Positive	Positive	Positive	Positive	–	–	–	–	–

Note: ^Δ^, In the ammonium chloride load test, it is a kind of test to check the function of distal renal tubular, urine pH > 5.3 after acidification indicates positive, which means that distal renal tubular acidification dysfunction caused type I RTA.

### Target region capture high-throughput sequencing and Sanger sequencing verification

Under the condition of ensuring sufficient coverage and depth of NGS sequencing, the probands of this family were subjected to high-throughput sequencing analysis of 6 gene target regions, including *SLC4A1*, *ATP6V0A4*, *CA2*, *SLC4A4*, and ATP6V1B1. The exon coding region and flanking regions of target genes were screened in detail, and copy number variation analysis was performed on all target genes. No major deletions or insertions were found in the patient (Table 2), and the mutant polymorphism (single-nucleotide polymorphisms (SNP)) locus with MAF (this value came from the population frequency information about dbSNP from the 1000 genomes project) greater than 1% was removed by filtration. Compared with the Clinvar database, 11 rare variants were screened, and no large deletions or duplications were found. A *ATP6V0A4* gene (NM-020632.2) exon16 variant associated with dRTA was detected, which is a missense variant heterozygote and named c.1631C > T (p.Ser544Leu) according to the HGVS international specification (http://www.hgvs.org/mutnomen/). This variant was not included in the ESP6500, dbSNP, and 1000G databases. Confirmed by Sanger sequencing (Fig. 1g, h), the amino acid at position 544 of exon 16 of the *ATP6V0A4* gene was changed from serine (Ser) to leucine (Leu) and the Clinvar database also has no relevant listings for this site (https://www.ncbi.nlm.nih.gov/clinvar/?term=ATP6V0A4%5Bgene%5D). Except for one unsampled case (I2), the other four patients (I2, II1, II9, and III2) carried the missense variant of p.S544L, and unaffected II5, III7, II10 (father of the proband), and III13 did not carry this variant. The family genetic linkage analysis showed that this variant had genetic co-segregation phenomenon. The PolyPhen-2 pathogenicity prediction score for the ATP6V0A4/p.S544L variant is 1 and the SIFT prediction score is 0. The variant is predicted to be harmful, which will cause a conformational change in the encoded protein and affect its normal function. Also Ser residues are often associated with phosphorylation. A mutation from Ser to Leu may affect protein function caused by loss of posttranslational modification^12^.

**Table 2:** Overview of screening for proband-related gene mutations in hereditary renal tubular acidosis family.

Gene	RefSeq	Nucleic acid alternation	Amino acid alternation	Mut site	Zygotic	Chromosome location	RS-ID	Maf	Mut_-_ type
ATP6V0A4	NM_020632	c.C1631T	p.S544L	exon16	Het	chr7	–	–	Mis
ATP6V1B1	NM_001692	c.C89T	p.T30I	exon1	Het	chr2	rs17720303	0.16	Mis
ATP6V1B1	NM_001692	c.C138T	p.S46S	exon2	Het	chr2	rs2266918	0.28	Syn
ATP6V1B1	NM_001692	c.C1002T	p.H953H	exon10	Hom	chr2	rs2072462	0.44	Syn
SLC4A4	NM_003759	c.C2859T	p.H604H	exon20	Hom	chr4	rs1453458	0.78	Syn
ATP6V0A4	NM_130840	c.T1812C	p.F554F	exon16	Hom	chr7	rs3807154	0.68	Syn
ATP6V0A4	NM_130840	c.C1662T	p.L87L	exon15	Hom	chr7	rs1026435	0.72	Syn
ATP6V0A4	NM_130840	c.T5C	p.V2A	exon2	Hom	chr7	rs10258719	0.68	Mis
CA2	NM_0012936	c.T259C	p.L87L	exon5	Het	chr8	rs703	0.58	Syn
SLC4A1	NM_000342	c.G1314A	p.S438S	exon12	Het	chr17	rs13306781	0.01	Syn
SLC4A1	NM_000342	c.A166G	p.K56E	exon4	Het	chr17	rs5036	0.06	Mis

Note: *RefSeq* NCBI reference sequence, *Mutation* *Mut*, *Het* heterozygote, *Hom* homozygous, *cytoBand* the chromosome segment in which the mutation is located, *Mis* missense mutation, *Syn* synonymous, *Nonfs ins* nonframeshift insertion, *FRE* frequency, *MAF* SNP minor allele frequency from dbSNP 1000 Genomes (population frequency information from the 1000 genomes project).

### *ATP6V0A4*/p.S544L gene mutant clone

**Fig. 2:**
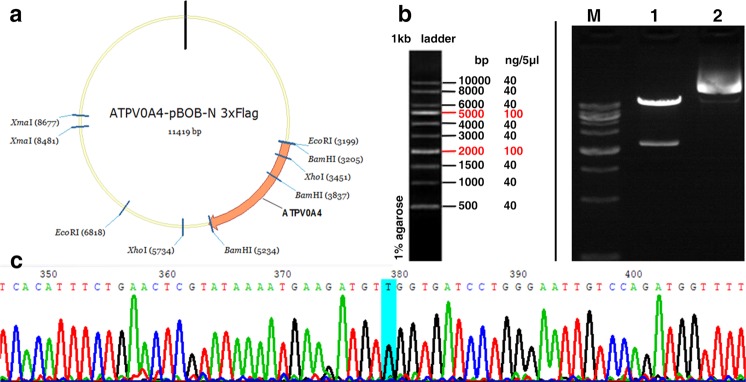
*ATP6V0A4*/p.S544L gene cloning. **a** *ATP6V0A4*/p.S544L mutant clone, schematic diagram of partial vector. **b** ATP6V0A4 wild type and *ATP6V0A4*/p.S544L mutant fragment by Xhol restriction enzyme digestion. **c** Successfully constructed vector sequenced verification.

In the present study, *ATP6V0A4* wild-type (-WT) and *ATP6V0A4*/p.S544L cloning vectors and eukaryotic expression vectors were successfully constructed. The structure of some vectors is shown in following Fig. 2a. The wild-type *ATP6V0A4* and the mutant *ATP6V0A4*/p.S544L (-mut) were digested by Xhol producing fragments approximately at 2000 and 5000 bp, which were consistent with the design (Fig. 2b). Successfully constructed vectors were verified by sequencing (Fig. 2c) and successfully transfected into HEK293T cells.

### Expression of *ATP6V0A4* gene and mutant in HEK293T cells and interaction with B1 subunit

**Fig. 3:**
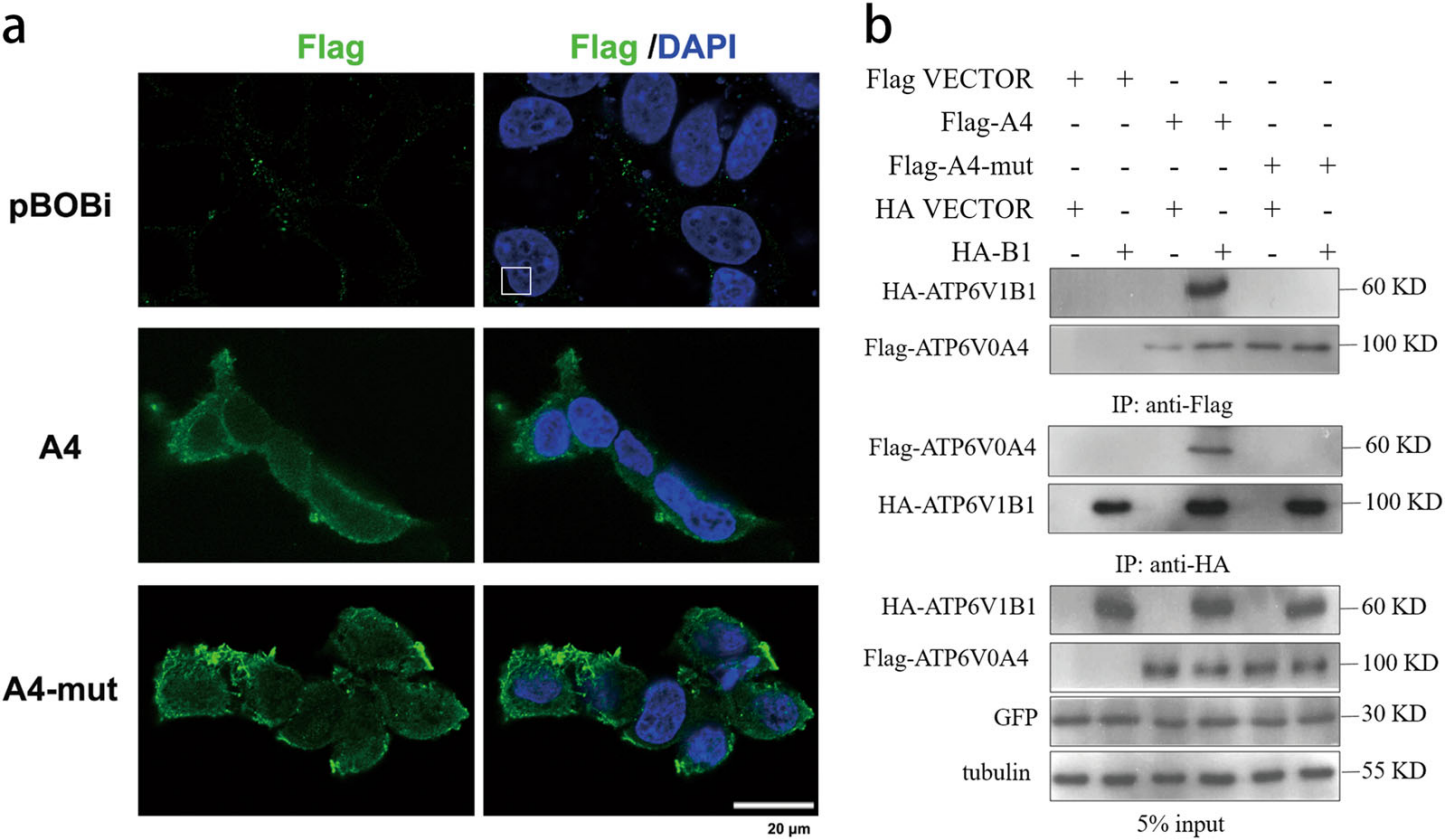
Expression of WT and mutant *ATP6V0A4* gene in HEK293T cells and interaction with B1 subunit. **a** Representative confocal images of pBOBi (empty-vector transfected) HEK293T cells (first row), or cells transiently transfected with A4 construct (second row), or A4-mut construct (third row). All panels show cells stained with anti-Flag antibodies (green). Nuclei are counter-stained with DAPI (blue). The results showed that both A4 and A4-mut are shown to be expressed on the cytoplasm, mostly on the cell membrane. The expression of A4-mut construct on the cell membrane increased, but the distribution was uneven. pBObi, empty vector control; A4, ATP6V0A4-wild type; A4-mut, *ATP6V0A4*/p.S544L mutant-type. **b** Confirmation of the interaction effect between A4, A4-mut and B1 subunits by Co-IP. HEK293T cells were transfected with Flag vector, or Flag-A4, or Flag-A4-mut, or HA vector, or HA-B1. 5% input was used as a positive control group, whole cell lysate was treated with tubulin antibody as internal reference and GFP antibody as external reference, and the loading and transfection effects were similar between the groups. Whole-cell lysate were separately immunoprecipitated with anti-FLAG antibody and anti-HA antibody. It showed that the expression levels between groups of *ATP6V1B1* were comparable, and the expression levels of A4 and A4-mut were comparable. Subsequently, both positive and negative immunoprecipitates were blotted and probed with anti-Flag antibody or anti-HA antibody, and B1 and a4 subunits were detected to bind to each other. When *ATP6V0A4* is mutated, B1 subunit cannot bind to mutated a4 subunit. Data shown are representative of three independent experiments. Flag-A4 Flag-tagged *ATP6V0A4*-wild typ, Flag-A4-mut Flag-tagged *ATP6V0A4*/p.S544L mutant-type, HA-B1 HA-tagged *ATP6V1B1*.

Immunofluorescence showed that both *ATP6V0A4*-WT and *ATP6V0A4*/p.S544L-mut proteins could be expressed on the cytoplasm, and most of them were expressed on the cell membrane. Compared with WT, we observed that the expression of *ATP6V0A4*/p.S544L-mut on the cell membrane increased and the distribution was not uniform, but the morphology of the cells did not change significantly (Fig. 3a). This indicates that the p.S544L-mut variant is retained in the cell membrane, and the mutation may affect the removal of this variant from the cell membrane. V-ATPase is a multi-subunit complex located in the B1 subunit of the V1 domain of the cytoplasmic V-ATPase. In the brush border membrane of α-ICs of the kidney, whether the V-ATPase is correctly assembled with the complete structure of the a4 subunit of the V0 domain on the membrane (the a4 subunit is encoded by the *ATP6V0A4* gene) affects the function of V-ATPase, thereby affecting the transport of cell membrane H^+^. To determine whether *ATP6V0A4*/p.S544L-mut affect the binding of the a4 subunit to the B1 subunit resulting in incorrect assembly of V-ATPase, an immunoprecipitation experiment was performed. Figure 3b shows representative immunoblots loaded with immunoprecipitate fractions that were pulled down with anti-Flag antibody or anti-HA antibody from lysates of HEK293T cells transfected with Flag-A4, or Flag-A4-mut, or HA-B1 or empty vector control. Results showed that the expression levels of *ATP6V1B1* among groups were comparable and the expression levels between *ATP6V0A4*-WT and -mut were comparable. Forward and reverse Co-IP were performed using anti-Flag or anti-HA antibodies. Results showed that a4 subunit-WT could bind to B1, while a4 subunit-mut could not bind to B1 subunit, which leads to incorrect assembly of the V1 and V0 domains, forming a structurally and functionally defective V-ATPase.

### ATPase activity assay (NADH absorbance change A340) in HEK293T cells carrying *ATP6V0A4*-WT and p.S544L-mut

**Fig. 4:**
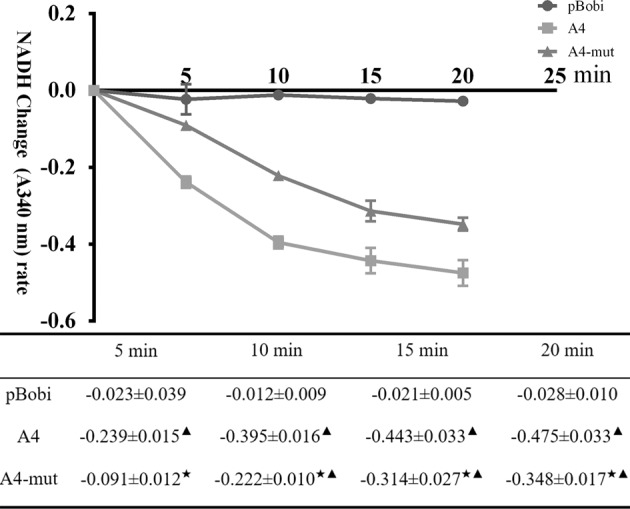
ATPase activity assay. ATPase activity was determined by NADH absorbance change (A340) in HEK293T cells carrying ATP6V0A4 wild type (A4) or ATP6V0A4/p.S544L-mut (A4-mut). In the form, the average change rate of NADH absorbance at different time periods ($\overline x$ ± SEM, *n* = 3), consistent with the ordinate data, calculated as NADH absorbance change (A340)/time. Black star < 0.01 vs. A4; black up-pointing triangle < 0.01 vs. blank vehicle control (pBobi).

During the period in 5, 10, 15, 20 min, *ATP6V0A4*/p.S544L-mut cells showed relatively weak ATPase activity [e.g., *ATP6V0A4*-WT (0.239 ± 0.015 A/min) vs. p.S544L-mut (0.091 ± 0.012 A/min), *P* < 0.05]. In the first 5 min, p.S544L-mut cells showed a decrease in slope compared with WT cells. Over time, the slope of the change in the absorbance of the ATPase NADH of p.S544L-mut cells was gradually consistent with that of WT cells (Fig. 4). The results show that the ATPase activity of p.S544L-mut carrier cells is weakened at an early stage. In order to maintain the overall cell function, it may be subsequently self-regulated and modified by endogenous pathways.

### PH fluorescence recovery assay in HEK293T cells carrying *ATP6V0A4*-WT and p.S544L-mut

**Fig. 5:**
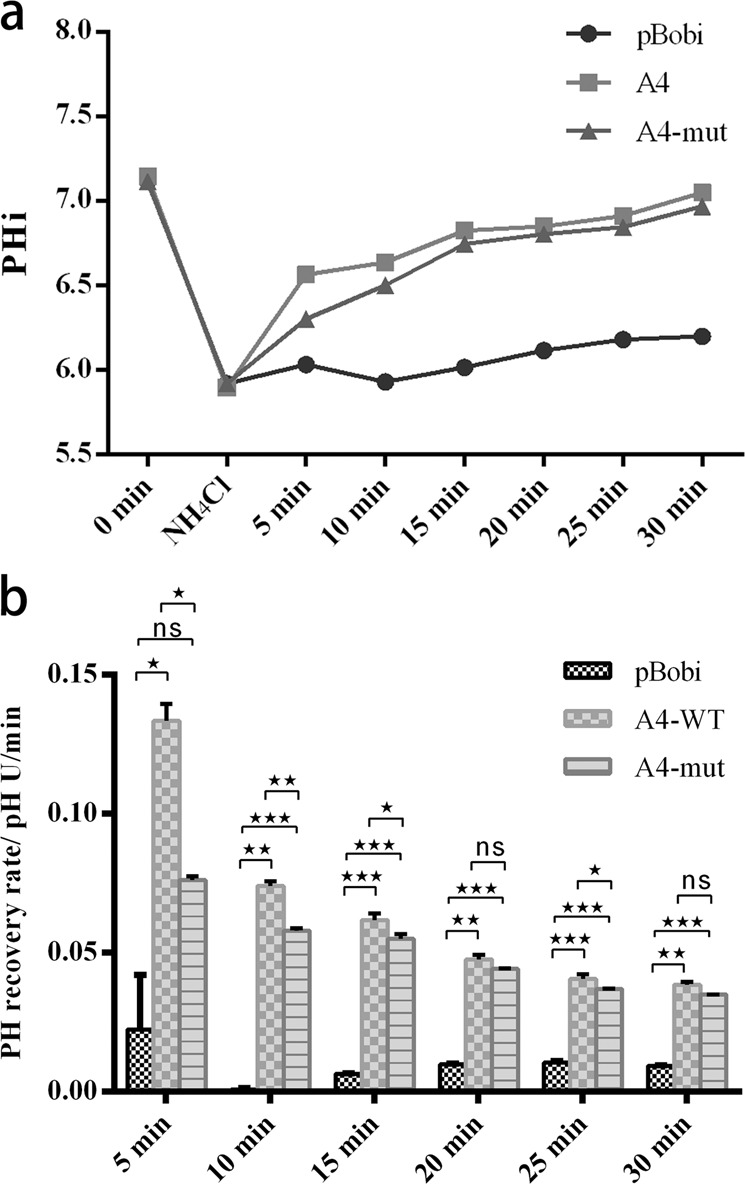
PH fluorescence recovery assay in HEK293T cells carrying *ATP6V0A4*-WT and p.S544L-mut. **a** Trends in PH levels at different time points after rapid acidification of cells in NH_4_Cl solution. **b** In HEK293T cells, the PH recovery rates were compared at different times after NH_4_Cl solution rapidly acidified cells. ns, no significance; Black star < 0.05, double black star < 0.01, triple black star < 0.001; pBObi empty-vector transfected, A4 *ATP6V0A4*-wild type, A4-mut *ATP6V0A4*/p.S544L mutant-type. Error bars indicate ±SEM.

The recovery rate of PH in different time periods after rapid acidification of NH_4_Cl solution was compared (Fig. 5), and it was found that the p.S544-mut mutant could affect the hydrogen ion transport function of V-ATPase. In HEK293T cells transfected with *ATP6V0A4*-WT, the cell pHi returned to 7.050 at 30 min after rapid acidification independent of sodium transport, and the average recovery rate of pHi was (0.0384 ± 0.0010) pH U/min. In HEK293T cells transfected with p.S544L-mut, the pH value returned to 6.967 after 30 min of rapid acidification without sodium transport, and the average recovery rate of pHi was (0.0349 ± 0.0001) pH U/min. The difference was not statistically significant (*P* > 0.05). In the 5, 10, 15, and 25 min after adding NH_4_Cl, the *ATP6V0A4*-WT group showed significantly higher recovery rate than the p.S544L-mut group, which was (0.1335 ± 0.0060) pH U/min vs. (0.0761 ± 0.0015) pH U/min in the first 5 min; (0.0740 ± 0.0017) pH U/min vs. (0.0580 ± 0.0008) pH U/min in the first 10 min; (0.0617 ± 0.0023) pH U/min vs. (0.0550 ± 0.0017 pH U/min in the first 15 min; (0.0406 ± 0.0016) pH U/min vs. (0.0370 ± 0.0000) pH U/min in the first 25 min (*P* < 0.05), and after rapid acidification of the cells in the NH_4_Cl solution, the pH recovery rate gradually became uniform over time. As described in the previous experiments, it was demonstrated that p.S544L-mut carrier cells may reduce the difference in the rate of PH recovery by endogenous mechanisms in order to maintain the overall cell function.

## Discussion

dRTA is a renal tubular disease with major defects in urinary acidification and acid excretion in the renal collecting duct system. The genetic form of dRTA has the characteristics of early onset age and serious disease, which seriously endangers human health, and the clinical manifestations usually begin in infancy or childhood^1,4,13^. With the development of molecular biology theory and sequencing technology, a variety of genes related to hereditary dRTA and their mutations have been reported, including at least three different gene mutations: *SLC4A1*, *ATP6V1B1*, *ATP6V0A4*, encoding exchanger AE1 of Cl^−^/HCO_3_^−^, B1 subunit and a4 subunit of V-ATPase, respectively^4,14,15^. More than 30 *ATP6V1B1* mutations and 40 *ATP6V0A4* mutations have been reported^16,17^, whereas China has only reported a few sporadic cases^18,19^. Mutations in *SLC4A1 (AE1)* are mainly inherited in an autosomal dominant manner, but also have autosomal recessive inheritance. The *ATP6V0A4* gene is located on chromosome 7q33–34 and has 23 exons, of which 20 exons encode a4 subunit containing 840 amino acids^20^. The *ATP6V1B1* and *ATP6V0A4* mutations affect two distinct subunits of V-ATPase: the B1 and a4 subunits, most of which are currently reported to be inherited in an autosomal recessive manner. However, patients with heterozygous *ATP6V1B1* and *ATP6V0A4* mutations, respectively had an increased risk of kidney stones and renal calcinosis in adulthood, or had incomplete dRTA^21,22^. Incomplete dRTA refers to insufficient uric acid in the renal collecting duct system of patients, but they usually have normal blood pH and bicarbonate, while acid provocation test reveals that uric acid deficiency does not cause urine pH below 5.3^23^. The patient with complete dRTA might exhibit mild clinical symptoms, such as mild metabolic acidosis or occasional kidney stones, or even severe clinical manifestations, such as severe metabolic acidosis, nephrocalcinosis, kidney stones, rickets, or osteomalacia, but preserved glomerular filtration rate. Except in the renal tissue, the B1 and a4 subunit genes of V-ATPase are also expressed in the epithelial cells of the endolymphatic sac (ES) in the cochlea^24^, which could explain progressive sensorineural hearing loss or abnormally enlarged partial vestibular aqueduct in most patients^8,25–28^. dRTA cases caused by *ATP6V1B1* mutations might develop deafness during early stages, while dRTA cases caused by *ATP6V0A4* mutations show mild or severe deafness or no deafness phenotype^29,30^. The difference in distribution and function of these two subunits may affect the heterogeneity of symptoms. Through genetic–phenotypic linkage analysis, we found that the family with p.S544L heterozygotes in the present study might be dominantly inherited, showing complete dRTA characteristics. Several studies have reported that several candidate genes are involved in hereditary dRTA, such as the Forkhead transcription factor Foxi1. The ultrastructure of the distal nephron of the kidney of Foxi1 knockout mice was changed. In the α-ICs of the collecting tube, the expression of various anion transporters, proton pumps and anion exchange proteins were deleted, accompanied by the deletion expression of Foxi1 in the inner ear and might be accompanied by sensorineural deafness^31^. Another example is the deletion of other V-ATPase subunits^32^, the chloride transporter SLC26A7^33^, the K-Cl co-transporter KCC4^34^, hensin/DMBT1^35^, Rhesus factor Rhcg^36^, etc.

The nephron cortical collecting duct (CCD) consists of chief cells that mediate Na^+^, K^+^, and water transport and ICs that are specifically used for acid–base transport, while ICs have two typical forms: acid-secreting α-ICs and HCO_3_^−^ secreting β-ICs. Chronic acidosis increases α-ICs and downregulates β-ICs, thereby increasing the net acid secretion of CCD^37^. The hydrogen secretion function of the distal renal tubule is mainly accomplished by α-ICs. In α-ICs, under the action of intracellular carbonic anhydrase II (CA type II), CO_2_ combines with H_2_O to form H_2_CO_3_, which then dissociates to form H^+^ and HCO_3_^−^. H^+^ is transported to the small lumen by V-ATPase located in the luminal side membrane of the α-ICs, while HCO_3_^−^ is transported back to the blood by AE1 located in the basement membrane. The H^+^ after secretion into the lumen combines with phosphate and NH_3_ in the lumen of the collecting duct^38^, and H^+^ combines with NH_3_ in the lumen of the collecting duct to form NH_4_^+^. The actively reabsorbed NH_4_^+^ dissociated to form H^+^ and NH_3_, while NH_3_ was dispersed into the lumen and H^+^ could be used as a substrate for V-ATPase. Therefore, the disorder of hydrogen secretion caused by mutations in the distal nephron may cause a decrease in the degree of acidification of the urine and a decrease in the secretion of NH_4_^[+3^. In addition, some transport factors are involved in the acid-secretion process of α-ICs, such as the K^+^–Cl^−^ cotransporter (KCC_4_) found in the α-ICs basolateral membrane of mice, where Cl^−^ outflow maintains the normal function of AE1 and mutations encoding these protein genes could result in dRTA in mice^8,34,39^.

V-ATPase is an important member of the ATP enzyme family. V-ATPase transfer protons relying on the energy produced by hydrolyzing ATP, which produces an electrochemical gradient across the membrane and regulates the pH inside and outside the cell. V-ATPase has played an important role in receptor-mediated endocytosis, protein degradation, storage of secreted proteins, intracellular membrane trafficking, absorption of Na^+^, renal acidification, bone resorption of osteoclasts, maturation of male sperm, etc. Many human diseases such as RTA, osteopetrosis, and tumor metastasis have important relationships with the physiological functions of V-ATPase^40^. V-ATPase consists of two functional parts, V1 and V0. The water-soluble spherical V1 is exposed on the surface of the membrane and has the function of catalyzing the hydrolysis of ATP and consists of eight subunits of A–H. The fat-soluble V0 is embedded in the membrane and is responsible for the transfer of protons and are composed of six subunits a, c, c’, c”, d, and e. The hexamer A3B3 composed of the A subunit and the B subunit in the V1 part is an energizing part, and it is connected to a peripheral rod (composed of C, E, G, H, a, and e subunits) to form a stator structure. The lipoprotein structure consisting of the subunits c, c’, and c” in the V0 moiety forms a rotor structure with the central rod (composed of subunits d, D, and F)^41,42^. The interaction between the subunits and the correct assembly are the key to maintaining the function of the V-ATP enzyme. For example, two proton hemichannels were formed inside a subunit towards the cytoplasm and the organelle cyst. When a proton in the cytoplasm enters the first proton hemichannel, it binds to a negatively charged glutamic acid residue on c, c’, or c”, while V1 decomposes the energy generated by ATP to drive the D and F subunit rotation. Then, the lipoprotein cyclic structure formed by c, c’, and c” is rotated. A neutral glutamic acid residue rotates with the lipoprotein loop, and when glutamate is rotated to another proton hemichannel, an arginine residue (R735) on the a subunit is interacts with the glutamate residue, lowering its pKa value to release protons, and the protons reach the other side of the membrane through the second proton hemichannel^43,44^. The a4 subunit may be involved in the assembly of V-ATPase, as well as the activity of ATPase and the transport of ions^45,46^. The a4 and B1 are subunit isoforms that occur together in the kidney^47^, both *ATP6V1B1* and *ATP6V0A4* mutations can cause dRTA. The mutations cause structural abnormalities in B1 or a4 may affect the binding of their two subunits, which in turn may hinder the correct assembly of V-ATPase. The free subunits may be localized to the brush border membrane, but the assembled V-ATPase is not sufficiently active, thereby reducing the ability of α-ICs to pump H^+^ into the lumen, leading to the development of dRTA^48^. Another example is the site at the carboxy terminus of a4 that binds to the glycolytic enzyme-phosphofructokinase, and mutations at this site could lead to dRTA^46^. In vitro experiments, cycloheximide chase assays revealed that V-ATPase a4 *R449H* were unstable relative to wildtype. Moreover, the a4 *R449H* variant of dRTA remained in the endoplasmic reticulum, and the cell surface expression was defective. a4 *R449H* increased the association with the V0 assembly factor VMA21 and decreased association with ATP6V1B1^49^. In addition, mutations in *ATP6V0A4* impair V-ATPase function, causing renal urinary acidosis, leading to bone softening due to loss of bone minerals (demineralization) and other causes. V-ATPase on the plasma membrane of osteoclasts plays an important role in bone resorption. Osteoclasts form a closed space around bone cells, and osteoclast V-ATPase pumps protons to the closed space to make the space an acidic environment, which causes the bone matrix to dissolve, activates secreted proteases, and promotes bone resorption^50^. Gene defects in V-ATPase in osteoclasts could also lead to recessive genetic disease such as osteopetrosis^51,52^.

This dRTA family has a distinctive feature, which is the incidence of five dRTA patients in the family. Mutation-linkage analysis suggests that the family has dominant inheritance characteristics. To our knowledge, the clinical features of human heterozygous *ATP6V0A4* mutants have not been well described to date, but in wild/*ATP6V0A4* deletion hybrid mice, high chloride metabolic acidosis has been demonstrated under chronic acid loading conditions^53^. In addition, *ATP6V0A4* p.S544L heterozygotes were found in a 40-year-old male patient with hypokalemic periodic paralysis, renal calcification, and alkaline urine incomplete dRTA confirmed by the NH4Cl load test, the furosemide-fludrocortisone loading test, and the HCO_3_^−^ loading test in Japanese population^21^, which is consistent with our findings in Chinese Han population. However, some patients in this study showed more severe complete dRTA. We also found no deafness phenotype among members of the family. These features and previous literature may indicate that the *ATP6V0A4* gene acts either dominantly or with recessive inheritance.
